# The effects of diet enhancement on the health of commercial bumblebee colonies

**DOI:** 10.1007/s13592-024-01132-1

**Published:** 2025-01-02

**Authors:** Rosaline A. Hulse, Annette Van Oystaeyen, Joanne D. Carnell, Danielle Beckett, William G. Grey, Dave Goulson, Felix Wackers, William O. H. Hughes

**Affiliations:** 1 School of Life Sciences, University of Sussex, Falmer, Brighton, UK; 2 Biobest Group NV, Ilse Velden 18, 2260 Westerlo, Belgium; 3 Department of Biology, University of York, York, UK

**Keywords:** *Bombus terrestris*, immunocompetence, commercial rearing, pollinator, nutrition

## Abstract

Commercially reared bees provide economically important pollination services for a diversity of crops. Improving their health is important both to maximise their pollination services and to avoid possible pathogen spillover or spillback with wild pollinators. Diet quality may directly or indirectly affect diverse aspects of bumblebee health, including colony development, individual size and immune health, but the impact of this remains unclear. Here we investigate experimentally the effect of diet quality during bumblebee colony development using three diets: (1) a standard pollen diet used in commercial rearing of colonies for sale, (2) an enhanced diet comprised of a wildflower pollen mix that was expected to be nutritionally superior (including by having an elevated protein content) and (3) a diet of pollen substitute mixed with the standard diet that was expected to be nutritionally poorer. The effect of diet quality on colony health was quantified using colony-level measures (colony weight, size [number of live workers] and number of dead individuals), and individual-level measures (body size, fat body size [proportion of body weight], total haemocyte count and phenoloxidase immune enzyme activity). Diet quality significantly affected colony growth, with colonies fed the enhanced diet growing larger and producing more reproductives than those fed either a standard or poor diet. The enhanced diet also resulted in bees that were significantly larger and had better immune health. The results show that diet can have important effects on the health of commercially reared bumblebees and suggest that the enhancement of standard-rearing diets may improve colony health.

**Supplementary Information:**

The online version contains supplementary material available at 10.1007/s13592-024-01132-1.

## Introduction

Pollinators are of fundamental ecological and economic importance. The majority of flowering plants are pollinated by insects and other animals, and plant-pollinator relationships are the drivers of much of the biodiversity seen in both wild and farmed terrestrial ecosystems (Ollerton 2017). Insects comprise the large majority of pollinators and therefore are vital to maintaining the health and function of these ecosystems, supporting the production of 75% of crop species and providing a global ecosystem service to food production of $215 billion every year (Vanbergen et al. 2013). Pollinator declines have been the focus of much recent attention, with pollinator health being threatened by a diversity of stressors, including natural and introduced diseases (Potts et al. 2010; Ollerton 2017; Botías et al. 2021; Khan et al. 2022). To compensate for wild pollinator populations often being unable to provide sufficient crop pollination services, managed bee populations have increasingly been used to provide this ecosystem service to agriculture (Aizen et al. 2009). As a result, several species of bumblebees (notably *Bombus terrestris* in Europe and *B. impatiens* in US), as well as the western honeybee, *Apis mellifera*, and some solitary bees (*Megachile rotundata*, *Osmia* spp.) are now commercially produced and transported at a global scale to support agricultural production of various fruit and vegetables (Delaplane and Mayer 2000; Velthuis and van Doorn 2006; Goulson and Hughes 2015).

The effectiveness of managed bees such as bumblebees as crop pollinators depends upon their health. Pollinator health is a multifaceted characteristic that includes both colony-level attributes, such as number of workers and food stores, and individual-level attributes, such as body size and immunocompetence (López-Uribe et al. 2020; Parreño et al. 2022). Healthier colonies, with more bees that are more active and more resistant to stresses such as parasites, will pollinate more flowers per unit time and thereby deliver a better ecosystem service to the crop. In addition, the commercial production, movement and introduction of high densities of managed insect species such as bumblebees involves the risk of introducing diseases or enhancing natural disease transmission, with negative consequences for conservation as well as commercial perspectives (Meeus et al. 2011; Goulson and Hughes 2015; Huang et al. 2015; Smith-Ramírez et al. 2023). Conservation and commercial interests are therefore aligned on the production of bumblebees that are, as far as possible, healthy and free of any parasite infections.

The potential for the bumblebee diet to support health is promising (Manson et al. 2010; Richardson et al. 2015; Koch et al. 2019). The quantity and quality of nutrition affect diverse aspects of bee health, including growth, flight activity and resistance to disease (Foley et al. 2012; Huang 2012; Roger et al. 2017). Some, but not all, studies suggest that more diverse diets support various components of bumblebee health (Tasei and Aupinel 2008; Baloglu and Gurel 2015; Moerman et al. 2016; Carnell et al. 2020). Although dietary diversity may help achieve this, it is the composition of macronutrients and micronutrients in the diet that affects health (Moerman et al. 2017). Protein, obtained by bumblebees solely from pollen, is of particular importance and is required for growth, longevity, immunocompetence and reproduction (Carnell et al. 2020). A lack of dietary protein negatively affects growth and constitutive immune function across bumblebee life stages (Brunner et al. 2014; Roger et al. 2017). The relative protein and lipid content of pollen varies between plant species and structures bee communities, with bumblebees regulating their pollen selection to achieve an optimum protein intake (Vaudo et al. 2016, 2024). Commercially produced bumblebees are fed pollen collected from free-flying honeybees, which may not have the optimum macronutrient ratios for bumblebees and is the primary route for transmission of pathogens into the bumblebee nests (Singh et al. 2010; Graystock et al. 2013; Goulson and Hughes 2015). Irradiation of pollen is now done by most producers to kill pathogens (Meeus et al. 2014; Graystock et al. 2016; Eakins et al. 2023) but adds to production costs and may affect the nutritional value of the pollen. Artificial diets for bumblebees could provide a standardised nutritional quality and eliminate the potential for pathogen exposure but have shown limited success to date (Bortolotti et al. 2020; Gekière et al. 2022).

Here we explore the relationship between nutrition and health in commercially produced bumblebees *B. terrestris audax*. We confirmed the colonies to be free of infection by the three main parasites, *Crithidia bombi*, *Vairimorpha* (*Nosema*) *bombi* and *V. ceranae*, using PCR (Table S1). We examined experimentally the effect of diet quality (enhanced, standard, poor) on bumblebee health at both the individual level (individual body size, fat body size [relative to size of abdomen], phenoloxidase and pro-phenoloxidase enzyme activity [PO and PPO], and total haemocyte count [THC]), and the colony level (colony weight, size [number of larvae, pupae and adults], the numbers of dead larvae and workers and the presence of reproductives). We tested the prediction that colonies fed better quality diets will grow larger, with more workers, and contain workers that are bigger and with a greater innate capacity to mount an immune response.

## Materials and methods

The experiment compared the effect of three proprietary diets developed by Biobest: a standard pollen diet used in commercial rearing, an enhanced pollen diet that was expected to be nutritionally superior, including by having a higher protein content, and a nutritionally poorer diet in which 40% of the pollen was replaced with a soy flour-based pollen substitute. The standard rearing diet comprised fresh frozen irradiated multifloral pollen that is used for feeding commercial colonies during production prior to their being sold (Biobest ID code Verkoop Groot/2016). This honeybee-collected pollen mix was a mixture of different European pollen species (spring-flowering and summer-flowering) from different suppliers and had an average protein content of 16% (measured using the Kjeldahl method by SGS, Belgium). The enhanced diet similarly comprised fresh frozen irradiated multifloral pollen, but from a different mixture of European pollen species, with an average protein content of 22% that has been shown to score the highest in Biobest’s in-house colony development assays and is part of a higher quality mixture fed to queen producing colonies (FW unpubl. data; Biobest ID code Kweek/2016). This honeybee-collected pollen mix was sourced from one supplier and consisted of a mix of spring-flowering plant species. The nutritionally poorer diet comprised a European soy flour-based pollen substitute developed by Biobest (ID code AD/2016) and mixed with the same pollen as in the standard diet at a ratio of 40:60. We used this because of its applied interest due to the potential benefits from the replacement of pollen with a substitute. Although pollen used in the enhanced and the standard and poor diets was known to differ in protein content, it is important to note that it may have also differed in other nutrients and any contaminants.

### Colony development and sample collection

Colonies of *B. terrestris audax* were reared from colony foundation on the three diets by the commercial producer Biobest, Westerlo, Belgium. Thirty colonies (ten for each diet) that were of similar size (15–20 workers) were shipped at 9 weeks from colony founding to the University of Sussex, UK. Upon arrival, the cardboard outer packaging was removed, and all colonies were kept for a further 4 weeks (to allow for staggered sample collection), in standardised laboratory conditions at 25–27 °C, 60–65% relative humidity and a 12-h light–dark cycle. In order to examine the effect of diet quality, rather than quantity, the colonies were limited to 30 g of the respective diet treatment each week (10 g/feed). Colonies were fed their respective diet treatment mixed with sugar water. Dry pollen was weighed, ground to a powder, mixed with 10–20% commercial sugar water solution (BIOGLUC®, Biobest) and made into pollen balls. Pollen balls were made 2 weeks in advance and stored at − 20 °C prior to use. The pollen substitute for the nutritionally poorer diet was supplied as a dough-like substance which was mixed with standard pollen in the same way as pollen balls were made.

Upon arrival at Sussex, the number of third and fourth instar larvae, pupae and adults was counted, and the presence of reproductives (adults and brood) and total colony weight were recorded. The collection of samples for body size analysis and immunological assays was staggered over 4 weeks. Each week, 10% of workers were removed from each colony, anaesthetised on ice for ~ 20 min and decapitated. Haemolymph was collected by inserting a microcapillary tube into the opening of the thorax and diluted 1:3 in PBS and stored at − 20 °C for total haemocyte counts (THC), or diluted 1:40 in sodium cacodylate, snap-frozen in liquid nitrogen (− 90 °C) and stored at − 80 °C for phenoloxidase (PO) and prophenoloxidase (PPO) analysis (Bocher et al. 2007; Negri et al. 2014). The gut was removed from the abdomen and stored in 1.5 mL of 100% ethanol at − 20 °C for pathogen screening, and the abdomen and intact fat body were kept dry and stored at − 20 °C for fat body analysis (see below). Thoraces and wings were stored in ethanol and kept for body size analysis based on wing marginal cell length. Images were taken with a Leica DFC295 camera and measured using ImageJ.

### Fat analysis

The insect fat body is the main site of antimicrobial peptide synthesis and as such is considered a key organ in insect immunity (Alaux et al. 2010; Arrese and Soulages 2010). Fat body size was measured for the bees collected (see above) by calculating their lipid content relative to their body size (Brown et al. 2000). At dissection, a small portion of the fat body was removed for pathogen screening along with the gut, with the majority of the fat body left intact on the inside of the abdomen. The abdomens were then dried at 70 °C for 5 days and then placed in diethyl ether for 24 h to dissolve lipids. The abdomens were rinsed in fresh diethyl ether before being re-dried at 70 °C for a further 5 days and reweighed. The difference between the first and second weight measurements was calculated as the fat content of the bumblebee and divided by the dry weight of the abdomen to obtain a ratio of fat content relative to body size for each worker.

### Phenoloxidase and pro-phenoloxidase activity

Phenoloxidase (PO) and its inactive precursor pro-phenoloxidase (PPO) are involved in the melanisation and encapsulation response of invertebrates upon exposure to pathogens (Gillespie et al. 1997; Lavine and Strand 2002). PO and PPO activity assays were carried out in microtiter plates prepared on ice (Bocher et al. 2007). Haemolymph samples of 15 µL were added to 5 µL of ddH_2_O for PO or 5 µL α-chymotrypsin (5 mg/mL in ice cold ddH_2_O) for PPO, together with 35 µL L-dopa (4 mg/mL in ice cold ddH_2_O). PO and PPO activity for each sample was performed simultaneously with treatments split across plates. Three technical replicates were performed per sample with negative controls for PO and PPO reactions on each plate. Prepared plates were placed immediately in a pre-heated microplate reader at 30 °C (Molecular Devices Spectra Max Plus 384). Reactions proceeded for 40 min with readings taken at 490 nm every 15 s. The slope during the linear phase of the reaction (*V*_max_) was taken as the enzyme activity value for further analyses.

### Total haemocyte count (THC)

Haemocytes are important cells in the insect immune system due to the roles they play in phagocytosis and encapsulation (Strand 2008; Alaux et al. 2010), and numbers have been shown in bumblebees to correlate with PO enzyme activity (Korner and Schmid-Hempel 2004). Haemolymph collected for THC was diluted 1:3 in PBS and stored at − 20 °C until processing, with 1 µL per bee used for counting. Haemocytes were incubated with DAPI (0.5ug/mL) on ice for 10 min, and cells were counted on a MACS Quant VYB FACS machine, for total DAPI positive cells per µl of haemolymph (Korner and Schmid-Hempel 2004).

### Pathogen screening

To confirm all workers were free from the disease, a *ca.* 0.3 cm^3^ tissue sample was dissected from each worker comprising the hindgut, Malpighian tubules and a small sample of fat body and placed in 100 µL DNA extraction solution (NaCl 100 mM, Tris pH8 10 mM, EDTA 25 mM, SDS 0.5%, 0.1 µg/µL proteinase K, 5% Chelex). DNA was extracted by overnight incubation at 55 °C followed by 20 min at 100 °C; the resulting solution was centrifuged at 2204 rcf for 1 h, and the supernatant was retrieved. An equal volume of isopropanol was added to the supernatant and gently agitated to mix. This solution was centrifuged at 2204 rcf for 1 h, and the supernatant was discarded. The remaining DNA pellets were washed once with 70% ethanol and allowed to air-dry for 20 min. Dry DNA pellets were resuspended in 100 µl molecular grade water for subsequent use at a 1:10 dilution in parasite screening PCR reactions. Bees were screened for the trypanosome *Crithidia bombi* and the microsporidians *Vairimorpha* (*Nosema*) *bombi* and *V. ceranae* using conventional PCR with parasite-specific primers and the host *18S* Apidae gene to control for DNA extraction quality (Table S1; Klee et al. 2006; Martín-Hernández et al. 2007; Meeus et al. 2010). Positive and negative controls were included on all plates. Positive control samples for pathogens were stock samples held in the lab from bees sampled locally and screened as part of other research projects. Amplicons were visualised on 3% agarose gels stained with ethidium bromide.

### Statistical analysis

We tested the effect of diet on each of the individual-level and colony-level health measures separately (Table S2). Individual measures of health were analysed using generalised estimating equations with individual bee ID included as a repeated measure within the colony to account for the structured nature of the data, and diet treatment and day of collection were included as fixed effects. Body size, PO and PPO were analysed using a gamma distribution and log link function, arcsine-transformed fat body as a proportion of body weight with a gamma distribution and identity link and THC with a negative binomial distribution and log link, using a quasi-likelihood model criterion to assess model fit. For the colony-level fitness measures, Q-Q plots and the deviance/df ratio were used to assess model fit. Colony weight and colony size were normally distributed and therefore investigated using general linear models with diet treatment as the explanatory variable. The number of dead workers and larvae was analysed using generalised linear models with negative binomial distributions and log link function, with diet treatment as the explanatory variable and colony size included as a covariate because larger colonies with more individuals were also more likely to have more dead individuals. The presence/absence of reproductives (adults or brood) was examined using a Fisher’s exact test. All analyses were performed in IBM SPSS 29.0 (IBM Corp., Armonk, NY). Multiple tests were controlled for the false discovery rate using the Benjamini–Hochberg procedure (Pike 2011; Menyhart et al. 2021).

## Results

### Pathogen screening

A total of 341 bumblebees were screened for pathogens across the 30 colonies. All were negative for the *C. bombi*, *V. bombi* and *V. ceranae* pathogens.

### Individual-level measures

**Figure 1.**
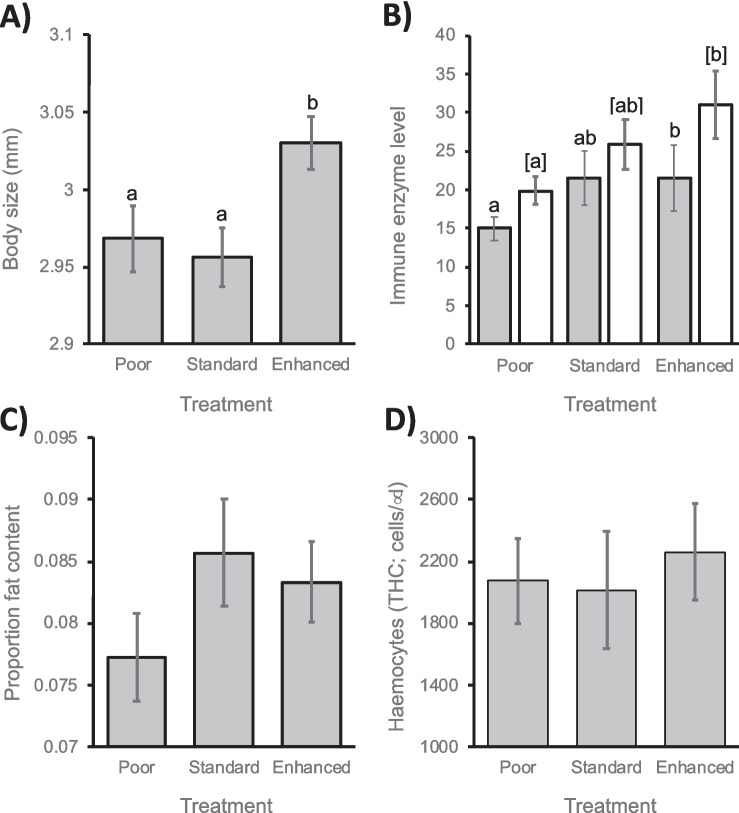
Individual-Level Measures of Bumblebee Health. The Effect of Diet on Mean ± S.E. **A** Body size, **B** Fat Body Size, **C** Phenoloxidase (PO; Grey) and Prophenoloxidase (PPO; white) Immune Enzyme Activity, and **D** Total Haemocyte Count (THC). *B. terrestris audax* Bumblebee Colonies Were Reared Under Laboratory Conditions from Colony Founding on One of Three Diets: a Standard Pollen Diet, an Enhanced Diet, and a Poor Diet (a Mixture of 40% Pollen Substitute with 60% Standard Diet). Letters Above Columns Indicate Treatments That Differed Significantly from Each Other at *p* < 0.05 in Pairwise Comparisons.

There was a significant effect of diet treatment on body size and PPO (respectively: *X*^2^ = 6.63, df = 2, *p* = 0.036; *X*^2^ = 6.9, df = 2, *p* = 0.032), but not on relative fat body size or THC (respectively: *X*^2^ = 2.99, df = 2, *p* = 0.224; *X*^2^ = 5.68, df = 2, *p* = 0.059), while the effect on PO was also nonsignificant when controlling for the false discovery rate (*X*^2^ = 6.21, df = 2, *p* = 0.045; Table S2). Bees fed the enhanced diet were larger and had higher levels of PPO than bees fed either the standard or poor diets (Figure 1).

### Colony-level measures

**Figure 2.**
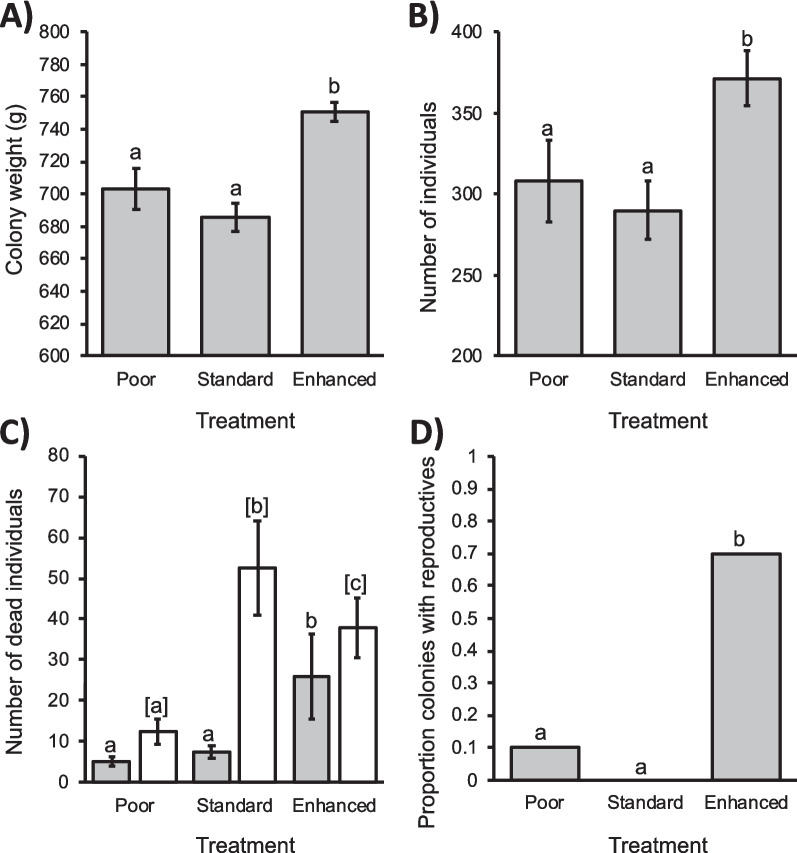
Colony-Level Measures of Bumblebee Health. The Effect of Diet on Mean ± S.E. **A** Colony Weight, **B** Colony Size (Total Number of Workers, Pupae and Larvae, Excluding Reproductive Adults and Brood), **C** Numbers of Dead Workers (Grey) and Dead Larvae (White), and the **D** Proportion of Colonies Producing Reproductives. *B. terrestris audax* Bumblebee Colonies Were Reared Under Laboratory Conditions from Colony Founding on One of Three Diets: a Standard Pollen Diet, an Enhanced Diet, and a Poor Diet (a Mixture of 40% Pollen Substitute with 60% Standard Diet. Counts Were Performed 9 Weeks After Foundation. Letters Above Columns Indicate Treatments That Differed Significantly from Each Other at *p* < 0.05 in Pairwise Comparisons.

There was a significant effect of diet treatment on colony weight, colony size, the numbers of dead workers and of dead larvae (when controlling for colony size) and the presence of reproductives (respectively, *F*_2,27_ = 12.7, *p* < 0.001; *F*_2,27_ = 4.37, *p* = 0.023; *X*^2^ = 15.2, df = 2, *p* = 0.001; *X*^2^ = 9.28, df = 2, *p* = 0.01; Fisher’s exact test *p* = 0.001). Compared to colonies fed the standard diet, colonies that received the enhanced diet treatment weighed ca. 10% more, had ca. 25% more individuals, had more dead workers but fewer dead larvae and were more likely to have already produced reproductives at nine weeks (Figure 2). Colonies that received the poor diet were not significantly different from those that received the standard diet on any measure except for the number of dead larvae, with the poor diet colonies having significantly fewer dead larvae than those receiving the standard diet (Figure 2D). Colony size had a significant effect on the number of dead workers, but not the number of dead larvae (Table S2).

## Discussion

Diet enhancement had significant effects on bumblebee health at both the individual and colony levels. At the individual level, diet enhancement positively affected bumblebee size and immunocompetence; at the colony level, it increased colony weight, size and the production of reproductives. These results provide evidence for the nutritional value of pollen for bumblebee health.

The positive effect of diet quality on body size is intuitive and follows on from existing work that has shown that adult worker size is determined by larval growth, which in turn is related to the quantity and nutritional quality of food that is provisioned to individual larvae (Ribeiro 1994; Pereboom 2000; Carnell et al. 2020). The positive effects of diet enhancement on the PO and PPO immune measures show that the beneficial effect of diet translates into improved immunocompetence. This finding supports previous work on individual bees that have shown they forage preferentially to support their own health, and that restricted diets can interfere with colony-level and individual-level development and susceptibility to disease (Génissel et al. 2002; Praz et al. 2008; Tasei and Aupinel 2008; Kitaoka and Nieh 2009; Somme et al. 2015; Moerman et al. 2016). It is also of applied relevance because it suggests that dietary enhancement will produce bees that are more resistant to any diseases they may encounter in factory or farm settings. The positive effect of diet enhancement on colony weight and colony size suggests that it provides significant additional benefits that could be due to the higher protein content of the enhanced diet or to other pollen components that may have differed between the diets. The higher number of dead workers in the enhanced diet could be due to workers having a shortened lifespan due to a high protein diet or a lifespan-fertility trade-off (Dussutour and Simpson 2012; Le Couteur et al. 2016) but may simply reflect the larger number of workers in colonies fed the enhanced diet. The use of soy flour in the nutritionally poorer diet means that it may have differed from the other diets in digestibility as well as protein content and other nutrients. However, the lack of a significant difference between the poor and standard diets for the majority of individual and colony-level measures suggests that substituting pollen with a pollen-replacement, at these proportions, can be no worse than the standard rearing diet. This may be an important consideration for commercial producers given that pollen costs are increasing by ~ 10% pa (FW unpubl. data) and that artificial diets will reduce the variability in diet quality and the potential for pathogens to enter the production process.

While the beneficial effect of enhanced diet for individual and colony health is clear, the implications for commercial producers may be more nuanced. The additional nutritional resources provided by the enhanced diet also resulted in more colonies producing reproductives, with all but one of the colonies that received the enhanced diet being already producing reproductives 9 weeks after foundation. The extra nutrition supports colony growth, resulting in the colony lifecycle progressing more quickly, with more workers being produced in a shorter period of time and the colony switching earlier to producing reproductives. Reproductives are of less value for crop pollination, so there is therefore a trade-off from a commercial perspective. This highlights the challenge of teasing apart the relationships and trade-offs between the many aspects impacted by individual and colony nutrition, including growth, reproduction, immunocompetence and longevity.

One limitation of our study was that in order to compare the nutritional quality of diets as opposed to quantity, pollen supply in the experiment was controlled at 30 g per week. This prevented colonies from regulating their nutrient intake. As colonies grow, their nutritional requirements increase commensurately, and the rate of colony development is not static but increases in line with available resources. Commercial colonies are provided with pollen ad libitum, while in natural colonies, colony growth will result in more foragers, thus increasing the nectar and pollen provision for the colony provided there are sufficient floral resources available. Larval ejection is a common colony-level response to stress (Goulson 2003), and the comparatively high levels seen in the standard colonies in our experiment are likely the result of these colonies increasing in size in line with food availability and then ejecting larvae due to nutritional limitation (Pomeroy and Plowright 1979; Génissel et al. 2002; Tasei and Aupinel 2008). This was not seen in the colonies on a poor diet, which had lower growth, fewer individuals and therefore lower nutritional requirements.

Diet and its quality are an important consideration for both commercial producers, who are motivated to provide a higher quality product, and also for conservation scientists looking to support the health of wild bee populations. The results here show that diet quality can have important direct and indirect effects on the health of bumblebees. This suggests that the health of commercially produced bumblebee colonies and individuals could be significantly improved by enhancing the diets used in their production. It also highlights the importance of high-quality floral resources throughout colony development to ensure the health of wild bees.

## Supplementary Information

Below is the link to the electronic supplementary material.

Supplementary file1 (DOCX 19 KB)

## Data Availability

The experimental data that support the findings of this study are available in Figshare with the identifier https://doi.org/10.25377/sussex.25459306.
