# Supplementary material for: The effects of diet enhancement on the health of commercial bumblebee colonies
Source: Apidologie. 2025 Jan 2;56(1):6. doi: 10.1007/s13592-024-01132-1 (PMC11695492; doi:10.1007/s13592-024-01132-1)
Supplement: Supplementary file 1 — Supplementary file1 (DOCX 19 KB) [file 13592_2024_1132_MOESM1_ESM.docx]

**Supplementary Materials**

**Table I.** PCR assay mixes and cycling conditions used to screen bumblebees for the three pathogens *Crithidia bombi*, *Vairimorpha* (*Nosema*) *bombi* and *V. ceranae*.

| **Primers & source** | **Assay mix** | | | | | | | | **Thermal cycling** | | | **Amplicon size (bp)** |
| --- | --- | --- | --- | --- | --- | --- | --- | --- | --- | --- | --- | --- |
|  | **dNTP (nM)** | **MgCl_2_ (nM)** | **5xbuffer (μl)** | **Taq (U)** | **Primer F (μM)** | **Primer R (μM)** | **Template (μl)** | **Total volume (μl)** | **1**  **Denaturing**  **Min \| Temp** | **2 Replication Sec \| Temp** | **3**  **Elongation Min \| Temp** |  |
| *Vairimorpha bombi*  (Klee et al. 2006) | 0.3 | 3.75 | 2 | 1.25 | 0.2 | 0.2 | 2 | 10 | 2 \| 95 | 35x  60 \| 95  60 \| 50  60 \| 72 | 4 \| 72 | 323 |
| *Vairimorpha ceranae*  (Martín-Hernández et al. 2007) | 0.2 | 1 | 2 | 1.25 | 0.2 | 0.2 | 1 | 10 | 5 \| 98 | 35x  15 \| 94  30 \| 58  45 \| 72 | 7 \| 72 | 218 |
| *Crithidia bombi*  (Meeus et al. 2010) | 0.2 | 1 | 2 | 1.25 | 0.2 | 0.2 | 1 | 10 | 2 \| 94 | 35x  30 \| 94  30 \| 56  45 \| 72 | 3 \| 72 | 420 |
| *Apidae* (internal)  (Meeus et al. 2010) | 0.2 | 1 | 2 | 1.25 | 0.2 | 0.2 | 1 | 10 | 5 \| 95 | 25x  15 \| 94  15 \| 56  30 \| 72 | 5 \| 72 | 130 |

**Table II**. Summary of statistical models used to investigate the effect of diet on colony-level and individual-level measures of bumblebee health, including dependent variables, explanatory factors and results for each model.

| **Measure of health** | **Statistical test and model parameters** | **Sig.** |
| --- | --- | --- |
| Colony weight | General linear model with diet treatment as explanatory variable | Diet *F_2,27_* = 12.7, *p* < 0.001 |
| Colony size (sum adults, larvae and pupae) | General linear model with diet treatment as explanatory variable | Diet: *F_2,27_* = 4.37, *p* = 0.023 |
| Number of dead workers | Generalised linear mixed model with a negative binomial distribution and log link. Diet treatment included as fixed effect (explanatory variable) and colony size as a covariate. | Diet *X^2^* = 15.2, df = 2, *p* = 0.001;  colony size *X^2^* = 1.42, df = 1,  *p* = 0.23 |
| Number of dead larvae | Generalised linear mixed model with a negative binomial distribution and log link. Diet treatment included as fixed effect (explanatory variable) and colony size as a covariate. | Diet *X^2^* = 9.28, df = 2, *p* = 0.01;  colony size *X^2^* = 0.042, df = 1,  *p* = 0.837 |
| Presence of reproductives (adults/brood) | Fisher’s Exact test | Diet *P* = 0.001; and between diet treatments, enhanced *P* = 0.003, and poor *P* = 1 |
| Body size | Generalised estimating equation with Gamma distribution and log link. Individual bee ID included as a repeated measure within colony; diet, day of collection and their interaction included as factors. | Diet *X^2^* = 6.63, df = 2*, p* = 0.036;  day of collection *X^2^* = 15.8, df = 8*, p* = 0.046; interaction: *X^2^* = 6.92, df = 11*, p* = 0.805 |
| Fatbody as proportion of body size | Generalised estimating equation of arcsine transformed data with gamma distribution and log link. Individual bee ID included as a repeated measure within colony; diet, day of collection and their interaction included as factors. | Diet *X^2^* = 2.99, df = 2*, p* = 0.224;  day of collection *X^2^* = 14.3, df = 8*, p* = 0.073; interaction *X^2^* = 17.7, df = 11*, p* = 0.089 |
| Total haemocyte count (THC) | Generalised estimating equation with negative binomial distribution and log link. Individual bee ID included as a repeated measure within colony; diet, day of collection and their interaction included as factors. | Diet *X^2^* = 5.68, df = 2*, p* = 0.059;  day of collection *X^2^* = 10.4, df = 8*, p* = 0.241; interaction *X^2^* = 6.58, df = 7*, p* = 0.474 |
| Phenoloxidase activity (PO) | Generalised estimating equation with Gamma distribution and log link. Individual bee ID included as a repeated measure within colony; diet, day of collection and their interaction included as factors. | Diet *X^2^* = 6.21, df = 2*, p* = 0.045;  day of collection *X^2^* = 0.686, df = 1*, p* = 0.407; interaction *X^2^* = 3.55, df = 2*, p* = 0.17 |
| Pro-phenoloxidase activity (PPO) | Generalised estimating equation with Gamma distribution and log link. Individual bee ID included as a repeated measure within colony; diet, day of collection and their interaction included as factors. | Diet *X^2^* = 6.9, df = 2*, p* = 0. 032;  day of collection *X^2^* = 0.347, df = 1*, p* = 0.556; interaction *X^2^* = 1.55, df = 2*, p* = 0.461 |
